# Admission Serum Ferritin Levels as a Predictor of Severe Disease in Hospitalized Pediatric Patients in Mexico

**DOI:** 10.1155/mi/8962790

**Published:** 2026-05-09

**Authors:** Grecia Abigayl Turrubiates-Hernandez, Jose Eduardo Mares-Gil, Carlos de la Cruz-de la Cruz, Oscar Tamez-Rivera

**Affiliations:** ^1^ Tecnologico de Monterrey, Escuela de Medicina y Ciencias de la Salud, Avenue Morones Prieto 3000, Monterrey, 64710, Nuevo León, Mexico, tec.mx; ^2^ Hematology Department, Facultad de Medicina y Hospital Universitario “Dr. Jose Eleuterio Gonzalez”, Universidad Autonoma de Nuevo Leon, Monterrey, Nuevo Leon, Mexico, uanl.mx; ^3^ Tecnologico de Monterrey, Institute for Obesity Research, Monterrey, Nuevo Leon, Mexico, tec.mx

**Keywords:** ferritin, predictor, severity

## Abstract

**Introduction:**

Biomarkers that predict the severity of a disease on admission would be a valuable tool for pediatricians. Ferritin has been proposed as a predictive biomarker for various conditions; however, multiple thresholds have been described depending on the diagnosis. A threshold for severe pediatric disease regardless of the final diagnosis is lacking.

**Material and Methods:**

We performed a single‐center retrospective observational study of patients admitted to the emergency department (ED) of a pediatric reference hospital in Mexico. Serum ferritin levels were requested to all admitted patients <16 years old and included in the study. Risk prediction models for various clinical outcomes were built using receiver operating characteristic (ROC) curves.

**Results:**

Admission ferritin levels were significantly higher in patients who required vasopressors (*p*  < 0.001), blood transfusion (*p*  < 0.001), and admission to the pediatric intensive care unit (PICU; *p*  < 0.001). Patients with admission ferritin >500 ng/mL had a longer hospital stay (*p* = 0.037). Admission ferritin was a predictor of all‐cause mortality (area under the curve [AUC] = 0.67, 95% confidence interval [CI]: 0.54–0.80, *p* = 0.009; sensitivity 55%, specificity 76.3%). A cut‐off point of 385 ng/mL was associated with mortality (OR 3.21, 95% CI: 1.29–7.97).

**Conclusion:**

We provide evidence that ferritin may be used as a predictive biomarker in the pediatric ED. In our study, ferritin levels on admission predicted all‐cause mortality. Multicenter studies are required to validate our findings in a larger sample.

## 1. Introduction

The use of biomarkers as predictive and prognostic tools for hospitalized patients has increased in recent years. Historically, the use of biomarkers in pediatrics has mainly focused on newborns and immunocompromised patients, leaving other pediatric groups as so‐called “biomarker orphans” [1]. Apart from the evidence obtained in neonatal studies, the majority of the available data on the use of biomarkers as predictive tools is extrapolated from studies in adult populations. The need for pediatric‐specific predictive biomarkers has long been recognized; however, the field of pediatrics faces many challenges as it comprises patients from different age groups and physiologic characteristics. Ferritin, known mainly for its role as an iron storage protein, is also an acute phase reactant that is secreted in several pro‐inflammatory conditions not only as a marker, but also as an effector during the host’s immune response against microorganisms. It has been observed that the increase in ferritin levels deprives bacteria from iron, thus, reducing bacterial load [2]. Theories of this elevation point towards an increase in the number and activity of macrophages, as well as due to the release of ferritin from damaged cells such as hepatocytes in cirrhosis or erythrocytes in cardiovascular or neoplastic diseases [3–5]. It has long been demonstrated that children and adults with serious infection, sepsis, and septic shock exhibit significantly elevated serum ferritin levels [6, 7]. But in spite of the immune role of ferritin during acute infection, a large body of evidence demonstrates significant association between serum ferritin levels and adverse clinical outcomes, even in populations with a high prevalence of iron deficiency [6–8]. In addition to its role during infection, ferritin has been used as a predictive tool in multiple clinical scenarios. Serum ferritin levels are effective indicators of disease activity in inflammatory and autoimmune conditions, such as juvenile idiopathic, hemophagocytic lymphohistiocytosis, and recently in multisystem inflammatory syndrome in children (MIS‐C) [2, 9]. Likewise, it has been described that elevated admission ferritin levels are associated with adverse clinical outcomes; however, myriad thresholds have been described depending on the studied condition and most studies have been performed in adult populations [10–14].

Having a biomarker that helps predict the clinical course from admission, regardless of the diagnosis, would be useful to identify high‐risk patients in the pediatric emergency department (ED), improve clinical outcomes, and reduce the economic burden of healthcare, particularly in low‐middle income countries. Ferritin is an accessible and widely available biomarker that may fulfill those needs; however, pediatric‐specific cut‐off values for severe disease are lacking. We aimed to explore the role of serum ferritin levels on admission among hospitalized pediatric patients as a predictor of severe disease, as well as their association with various clinical outcomes.

## 2. Materials and Methods

### 2.1. Study Design

This is an observational and retrospective single‐center study.

### 2.2. Study Population

Previously healthy pediatric patients younger than 16 years admitted to Hospital Regional Materno Infantil (HRMI) in Monterrey, Mexico, from August 2021 to August 2022, and in whom serum ferritin levels were obtained on admission to the ED as part of the standard of care were studied. Data from patients who were referred to another institution, received blood transfusion 2 months prior, received iron supplementation 6 months prior, and had a previous hematological diagnosis (i.e., acute or chronic leukemia, lymphoma, thalassemia, and Langerhans cell histiocytosis) were excluded.

### 2.3. Data Collection

Sociodemographic, clinical, and laboratory data from the electronic clinical chart were reviewed. The clinical outcomes of interest included: prolonged hospital stay (≥6 days), admission to the pediatric intensive care unit (PICU), use of invasive mechanical ventilation, vasopressor support, blood transfusion therapy, and mortality. Admission serum ferritin levels were dichotomized as normal or elevated, considering the age‐based upper limit established by the American Academy of Pediatrics (AAP) [15] (Annex 1). Patients were stratified in three groups according to their age (Group 1: <6 months; Group 2: 6 months to <5 years; Group 3: 5–16 years). Patients with elevated ferritin levels were further classified as having mild (<500 ng/mL), moderate (500–1000 ng/mL), and severe (>1000 ng/mL) ferritinemia. Hemoglobin, mean corpuscular volume (MCV), and mean corpuscular hemoglobin (MCH) values were classified as normal or decreased based on age‐specific ranges established in international literature [15, 16].

### 2.4. Statistical Analysis

Basic descriptive statistics were used to describe sociodemographic characteristics. Categorical data was reported as frequencies and percentages, while continuous data was reported as means and standard deviations (SDs), or medians and interquartile ranges (IQRs), depending on their distribution. Distribution of numerical variables was assessed using the Kolmogorov–Smirnov *Z*‐test. Categorical variables were compared with the chi‐square test or Fisher’s exact test, depending on their distribution. Numerical variables were compared with the Mann–Whitney *U* test. Correlation between variables was analyzed with Spearman’s rank correlation coefficient. Receiver operating characteristic (ROC) curves were used to calculate the area under the curve (AUC) of different possible mortality predictors. In those with an acceptable AUC, the best cut‐off point was obtained using the Youden index. The sensitivity and specificity of said cut‐off points were calculated. Subsequently, a 2 × 2 contingency table was constructed, categorizing patients according to the selected ferritin cut‐off and outcome status, from which odds ratios were calculated to estimate the strength of association between elevated ferritin levels and mortality based on the odds of the event occurring in each group. Given the limited number of outcome events, multivariate analysis was not performed, as the data did not meet the events‐per‐variable rule of thumb required to ensure stable and reliable estimates and to avoid model overfitting. The selection of the cut‐off point was prespecified and based on ROC curve analysis using the Youden index, rather than being arbitrarily defined. The *p*‐value was set at <0.05 and a 95% confidence interval (CI) was considered statistically significant. Statistical analyses were performed using SPSS software v25 (IBM SPSS Statistics, IBM Corporation, Armonk, NY, USA.)

## 3. Results

### 3.1. Demographic Characteristics

A total of 391 subjects were included. Most patients were male (51.4%). Median age was 1.9 years (IQR 0.5–6.3). Age distribution according to age groups was as follows: 93 patients (23.8%) were <6 months old, 182 (46.5%) were between 6 months and <5 years, and 116 (29.7%) were between 5 and 16 years.

### 3.2. Baseline Characteristics

The most common diagnoses were infectious diseases (71.9%), followed by rheumatologic diseases (12.3%). Median serum ferritin on admission was 155.3 ng/mL (IQR = 78.8–385.8). More than half (56%) of the patients had elevated age‐based serum ferritin levels on admission to the ED. Of the total sample, 317 patients (81.1%) had mild hyperferritinemia (<500 ng/mL), 38 (9.7%) had moderate hyperferritinemia (500–1000 ng/mL), and 36 (9.2%) had severe hyperferritinemia (>1000 ng/mL). Median CRP was 1.48 mg/L (IQR = 0.19–6.9), and median hemoglobin was 12 g/dL (IQR = 10.7–13). Of the total sample, 114 patients (29.2%) were anemic according to the AAP’s age‐based reference ranges (9). Twenty‐four patients (6.1%) had microcytic hypochromic anemia. Admission to the PICU was necessary in 35 patients (9%). Twenty patients (5%) died during their hospitalization. Table 1 describes additional information regarding clinical outcomes.

**Table 1:** Clinical outcomes of pediatric patients hospitalized in a reference center in Northeastern Mexico.

Variable	*n* = 391
Days of hospital stay, median (IQR)	5 (1–8)
Prolonged stay, *n* (%)	150 (38.4)
Use of vasopressors, *n* (%)	31 (7.9)
Admission to PICU, *n* (%)	35 (9)
IMV, *n* (%)	34 (8.7)
Transfusion therapy, *n* (%)	15 (3.8)
Death, *n* (%)	20 (5.1)

Abbreviations: IMV, invasive mechanical ventilation; IQR, interquartile range; PICU, pediatric intensive care unit.

### 3.3. Main Findings

We did not find an association between dichotomized admission serum ferritin levels (normal or increased) and the studied clinical outcomes (Table 2). Interestingly, a greater use of vasopressors (*p*  < 0.001), admission to the PICU (*p*  < 0.001), invasive mechanical ventilation (*p*  < 0.001), and transfusion of blood products (*p*  < 0.001) was observed in those with higher serum ferritin levels (Table 3). Patients with admission serum ferritin levels >500 ng/mL had a more prolonged median hospital stay (5 days), compared to those with ferritin <500 ng/mL (4 days; *p* = 0.037). Likewise, a higher mortality was observed in patients with higher serum ferritin levels (*p*  < 0.001; Table 3).

**Table 2:** Dichotomized serum ferritin levels (normal and increased) according to the type of diagnosis and outcomes of pediatric patients hospitalized in a reference center in Northeastern Mexico.

Variable	Total *n* = 391	Increased^a^ *n* = 219	Normal^a^ *n* = 172	*p*
Diagnosis type, *n* (%)				0.135
Infectious	281 (71.9)	152(69.4)	129 (75)	
Rheumatologic	48 (12.3)	31 (14.2)	17 (9.9)	
Endocrinologic	6 (1.5)	3 (1.4)	3 (1.7)	
Respiratory	7 (1.8)	7 (3.2)	0 (0)	
Musculoskeletal	3 (0.8)	1 (0.5)	2 (1.2)	
Neurologic	23 (5.9)	15 (6.8)	8 (4.7)	
Gastrointestinal	7 (1.8)	4 (1.8)	3 (1.7)	
Other	16 (4.1)	6 (2.7)	10 (5.8)	
Days of hospital stay, median (IQR)	5 (1–8)	5 (1–8)	5 (1–8)	0.652
Prolonged stay, *n* (%)	150 (38.4)	82 (37.4)	68 (39.5)	0.673
Use of vasopressors, *n* (%)	31 (7.9)	20 (9.1)	11 (6.4)	0.32
Admission to PICU, *n* (%)	35 (9)	17 (7.8)	18 (10.5)	0.226
IMV, *n* (%)	34 (8.7)	19 (8.7)	15 (8.7)	0.987
Transfusion therapy, *n* (%)	15 (3.8)	11 (5)	4 (2.3)	0.168
Death, *n* (%)	20 (5.1)	14 (6.4)	6 (3.5)	0.196
Admission to PICU and death	11 (2.8)	6 (2.7)	5 (2.9)	0.578

Abbreviations: IMV, invasive mechanical ventilation; IQR, interquartile range; PICU, pediatric intensive care unit.

^a^Age‐adjusted values according to the AAP.

**Table 3:** Association of degree of hyperferritinemia at admission (mild, moderate, and severe) with the type of diagnosis and outcomes of pediatric patients hospitalized in a reference center in Northeastern Mexico.

Variable	Mild (<500) *n* = 317	Moderate (500–1000) *n* = 38	Severe (>1000) *n* = 36	*p*
Diagnosis type, *n* (%)				0.763
Infectious	220 (69.4)	30 (78.9)	31 (86.1)	
Rheumatologic	39 (12.3)	5 (13.2)	4 (11.1)	
Endocrinologic	5 (1.6)	1 (2.6)	0 (0)	
Respiratory	7 (2.2)	0 (0)	0 (0)	
Musculoskeletal	3 (0.9)	0 (0)	0 (0)	
Neurologic	21 (6.6)	1 (2.6)	1 (2.8)	
Gastrointestinal	7 (2.2)	0 (0)	0 (0)	
Other	15 (4.7)	1 (2.6)	0 (0)	
Days of hospital stay, median (IQR)	4 (1–8)	5 (3–12)	5 (1–14)	**0.037**
Prolonged stay, *n* (%)	117 (36.9)	18 (47.4)	15 (41.7)	0.416
Use of vasopressors, *n* (%)	16 (5)	3 (7.9)	12 (33.3)	**<0.001**
Admission to PICU, *n* (%)	18 (5.7)	8 (21.1)	9 (25)	**<0.001**
IMV, *n* (%)	16 (5)	6 (15.8)	12 (33.3)	**<0.001**
Transfusion therapy, *n* (%)	7 (2.2)	1 (2.6)	7 (19.4)	**<0.001**
Death, *n* (%)	8 (2.5)	1 (2.6)	11 (30.6)	**<0.001**
Admission to PICU and death	5 (1.6)	1 (2.6)	5 (13.9)	**<0.001**

*Note*: Bold values indicate statistical significance (*p* < 0.05).

Abbreviations: IMV, invasive mechanical ventilation; IQR, interquartile range; PICU, pediatric intensive care unit.

**Figure 1:**
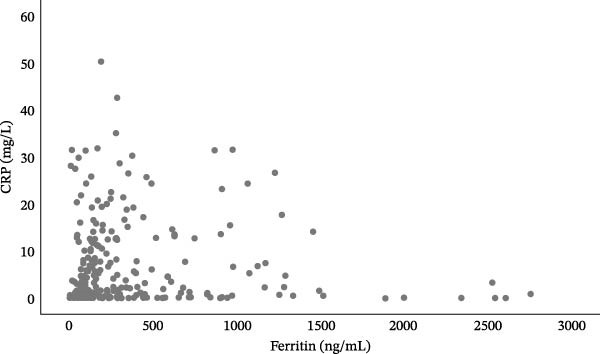
Correlation between serum ferritin levels and CRP in pediatric patients hospitalized in a reference center in Northeastern Mexico.

Of the 391 patients included in the study, 359 (91.8%) also had admission serum CRP levels. Of those, 143 subjects (39.8%) had elevated age‐based CRP and ferritin levels, 152 (42.3%) had at least one of them elevated and 64 subjects (17.8%) had both values within normal ranges. A significant correlation was observed between admission serum ferritin levels and admission CRP (rho = 0.2, *p*  < 0.001; Figure 1). No significant differences in mortality were found between the three groups (*p* = 0.457).

Patients who died during their hospitalization had significantly higher admission serum ferritin levels (414 ng/mL, IQR = 103.7–2255) compared to those who survived (151.7 ng/mL, IQR = 77.8–355.1 ng/mL; *p* = 0.009). Similarly, we found higher levels of CRP in patients who died (14.2 mg/L, IQR = 0.2–21.7) compared to those who survived (1.3 mg/L, IQR = 0.1–6.6; *p* = 0.022).

**Figure 2:**
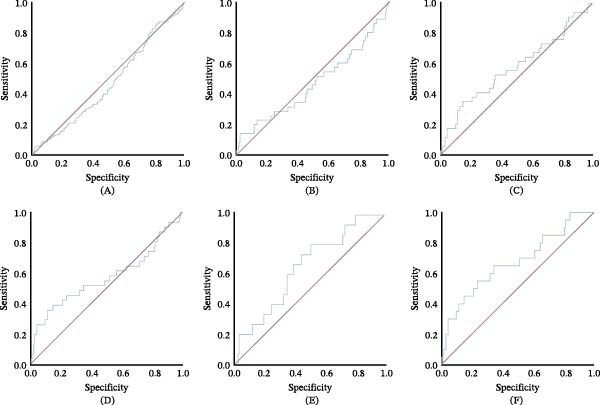
ROC curves of serum ferritin levels for the prediction of (A) prolonged hospital stay; (B) admission to PICU; (C) invasive mechanical ventilation; (D) use of vasopressors; (E) transfusion of blood products; (F) death during the hospital stay from any cause.

We found that admission serum ferritin levels were an acceptable predictor of all‐cause mortality during the hospital stay (AUC = 0.673, 95% CI: 0.54–0.805; *p* = 0.009) with a sensitivity of 55% and specificity of 76.3% in patients of any age (Figure 2). Furthermore, a serum ferritin cut‐off point of 385 ng/mL was associated with a higher probability of death (OR 3.21, 95% CI: 1.29–7.97; Table 4).

**Table 4:** Diagnostic performance of admission serum ferritin levels for predicting clinical outcomes.

Outcome	AUC	95% CI	*p*	Sensitivity	Specificity
All‐cause mortality during hospital stay^a^	0.673	0.540–0.805	0.009	55%	76.3%
Prolonged hospital stay	0.469	0.411–0.528	0.305	–	–
Admission to PICU	0.467	0.355–0.580	0.526	–	–
Use of invasive mechanical ventilation	0.577	0.465–0.688	0.140	–	–
Use of vasopressor support	0.571	0.444–0.698	0.191	–	–
Need for blood transfusions	0.645	0.515–0.776	0.057	–	–

Abbreviations: AUC, area under the curve; CI, confidence interval; CRP, c‐reactive protein; PICU, pediatric intensive care unit.

^a^Sensitivity and specificity analyses were performed exclusively for all‐cause mortality during hospital stay, given that it was the only outcome demonstrating statistically significant predictive performance. The optimal ferritin cut‐off value was 385 ng/mL.

Admission serum ferritin levels were not a predictor of prolonged hospital stay (AUC = 0.469, 95% CI: 0.411–0.528, *p* = 0.305), admission to PICU (AUC = 0.467, 95% CI: 0.355–0.58, *p* = 0.526), use of invasive mechanical ventilation (AUC = 0.577, 95% CI: 0.465–0.688, *p* = 0.14), use of vasopressor support (AUC = 0.571, 95% CI: 0.444–0.698, *p* = 0.191) or need of blood transfusions (AUC = 0.645, CI: 95% 0.515–0.776, *p* = 0.057; Figure 2). Regarding CRP, an AUC of 0.656 (95% CI: 0.509–0.804) was found for the prediction of mortality. The best CRP cut‐off point, with the highest Youden index, was 14.17 mg/L, with a sensitivity of 52.6% and specificity of 88.3%.

## 4. Discussion

### 4.1. Overview

The role of ferritin as an acute phase reactant has long been recognized; however, its use as a predictive tool for clinical outcomes in pediatric patients is scarce. Furthermore, previous pediatric studies that have explored the predictive role of ferritin levels have focused on specific diagnoses, including sepsis and MIS‐C. We aimed to assess the capacity of admission ferritin levels to predict relevant clinical outcomes among 391 hospitalized pediatric patients in Mexico from August 2021 to August 2022.

### 4.2. Comparison With Previous Studies

In our study, median serum ferritin was 155.3 (78.8–385.8) ng/mL; 56% of the patients had elevated age‐specific serum ferritin levels on admission to the ED. This is consistent with a previous study by Tonial et al. [17], who reported a median serum ferritin of 150.5 ng/mL in pediatric patients admitted to the PICU. As expected, due to the high variability of ferritin, other studies in similar populations have reported higher median serum ferritin levels, even double or triple from ours [7, 8].

Notably, the limited available studies that have explored the predictive use of ferritin in children are lacking age‐specific analyzes. This is significant because, as discussed earlier, normal ferritin levels vary according to age. For example, a ferritin level of 700 ng/mL is considered normal in a 2‐week‐old infant. Given this variability and considering the regulatory pathways, it would be reasonable to expect that there would be age‐related thresholds for ferritin as an inflammatory marker as well. We theorized that the clinical value of serum ferritin should be adjusted to the patient’s age, and thus, we included analyzes depending on different age‐ranges.

We found that age‐specific [15] elevated ferritin levels were not associated with the type of diagnosis. Interestingly, when stratifying according to the degree of ferritinemia, we found an association between severe hyperferritinemia on admission (>1000 ng/mL) and the use of vasoactive drugs (*p*  < 0.001), admission to the PICU (*p*  < 0.001), mechanical ventilation (*p*  < 0.001), blood transfusion (*p*  < 0.001), and death (*p*  < 0.001). We also observed a longer hospital stay in patients with a higher level of serum ferritin on admission. Previous studies that stratified ferritin levels in quartiles did not find an association with the need for vasoactive drugs or mechanical ventilation [17]. To our knowledge, no previous study has documented an association with prolonged hospital stay and high ferritin levels in children.

Overall mortality in our study was 5.1%, ranging from 2.5% to 2.6% for those with mild and moderate hyperferritinemia, and significantly increasing to 30.6% in patients with severe hyperferritinemia. A similar discovery has been previously reported in a comparable population where a tenfold increase in serum ferritin was associated with a fivefold increase in mortality [17]. Likewise, Ghosh et al. [8] found a similar association in a pediatric population diagnosed with septic shock, despite the fact that two thirds of the population had hypochromic microcytic anemia.

Historically, numerous cut‐off values of ferritin for predicting clinical outcomes have been suggested, most of them >500 ng/mL. For example, among children with sepsis in a resource‐limited setting, ferritin levels >500 ng/mL were associated with 58% mortality after adjusting for severity of presentation, with 64% sensitivity and 80% specificity for predicting death [7]. Although 500 ng/mL has been consistently used as a predictive cut‐off value, previous studies have highlighted the need to establish lower serum ferritin thresholds to define pediatric hyperferritinemia [8, 17]. For instance, Horvat et al. [18] found that a ferritin cut‐off value of 373 ng/mL was associated with higher mortality in hospitalized patients, regardless of the diagnosis (OR 14.0 [2.7–72.6]; *p* = 0.002). Our findings are consistent with these conclusions, as we found that admission serum ferritin was a predictor of in‐hospital all‐cause mortality independently of age and diagnosis. Notably, patients with a ferritin cut‐off value of 385 ng/mL had 3.2 times more probability of dying. We support the need to reconsider the commonly used ferritin cut‐off value of 500 ng/mL, as ferritin and other biomarkers are known to behave differently among different age groups. Using a lower ferritin threshold would aid in the early detection of potentially deteriorating patients from admission to the ED, thus reducing adverse clinical outcomes including mortality.

The precise mechanisms resulting in the association between CRP and ferritin with mortality are unclear, and it is possible that elevations in both would reflect activation of different inflammatory pathways. We observed a discretely significant correlation between serum ferritin levels and CRP (rho = 0.2, *p*  < 0.001). Further studies are required in order to analyze the role of combining ferritin and CRP levels for predicting clinical outcomes.

### 4.3. Clinical Implications

Ferritin is an inexpensive, widely available, and low‐complexity test that can be easily performed in most clinical laboratories—even those without access to advanced diagnostic technologies. This makes it especially valuable in low‐resource or underserved healthcare environments, where timely risk stratification and early intervention are critical. Using admission ferritin levels as a predictor of serious disease and mortality in children would aid in the early detection of potentially deteriorating patients from admission to the ED, thus, reducing adverse clinical outcomes including mortality. It is worth noting that although nutritional anemia is highly prevalent in Mexico, only 6.1% of our patients had iron deficiency anemia. This is important as ferritin might not behave as expected among patients with nutritional anemia; however, this does not seem to be a limitation in our analyses. Our study reduces the gap of knowledge regarding the use of ferritin as a predictive biomarker for adverse health outcomes in children. We encourage the broader use of ferritin testing as part of early clinical evaluation protocols, particularly in contexts where other laboratory or imaging resources are limited. Further multicenter prospective studies with larger samples are required in order to replicate our findings.

### 4.4. Limitations

This study has several limitations that should be acknowledged. First, due to the exploratory design and the limited number of outcome events—particularly mortality—it was not feasible to perform a multivariate analysis that met the recommended events‐per‐variable rule of thumb. As a result, we were unable to develop a robust multivariable or predictive model to adjust for potential confounders, which limits causal inference. Additionally, the sample size and the relatively low frequency of some clinical outcomes may have reduced the statistical power to detect more subtle associations. Despite these limitations, our findings provide valuable preliminary evidence supporting the role of ferritin as a potential early marker of disease severity, and should be interpreted as hypothesis‐generating for future studies with larger sample sizes and adequate event rates.

## 5. Conclusions

Admission serum ferritin levels predicted severe disease and adverse clinical outcomes in hospitalized pediatric patients. A greater use of vasopressors, admission to the PICU, invasive mechanical ventilation, use of blood transfusion, and death were found among patients with higher levels of serum ferritin. Patients with admission serum ferritin >500 ng/mL had a longer hospital stay. We found that admission serum ferritin is an acceptable predictor of all‐cause mortality during hospitalization, and that a serum ferritin cut‐off value of 385 ng/mL confers 3.2 times greater probability of death. Ferritin could be used as part of early assessment protocols, especially in resource‐limited settings. Further studies are required in order to replicate our findings among pediatric populations.

## Author Contributions

Grecia Abigayl Turrubiates‐Hernandez contributed to data collection, analysis and interpretation of data, and manuscript submission. Jose Eduardo Mares‐Gil contributed to data collection and critical review for important intellectual content. Carlos de la Cruz‐de la Cruz contributed to data analysis, critical review for important intellectual content and final approval of the version to be submitted. Oscar Tamez‐Rivera contributed to conception of the idea and design of the study, interpretation of data, drafting, and final approval of the version to be submitted.

## Funding

No funding was received for this manuscript.

## Ethics Statement

The study protocol followed the principles of the Helsinki Declaration and was approved by the Institutional Review Ethics and Research Board at the hospital. Informed consent was waived due to the retrospective nature of the study design.

## Conflicts of Interest

The authors declare no conflicts of interest.

## Supporting Information

Additional supporting information can be found online in the Supporting Information section.

## Supporting information


**Supporting Information** Annex 1: Serum ferritin levels reference range according to age and sex. Modified from Soghier et al. [15].

## Data Availability

The data are available upon request from corresponding author.
