# Supplementary material for: Admission Serum Ferritin Levels as a Predictor of Severe Disease in Hospitalized Pediatric Patients in Mexico
Source: Mediators Inflamm. 2026 May 9;2026:8962790. doi: 10.1155/mi/8962790 (PMC13157303; doi:10.1155/mi/8962790)
Supplement: Supplementary file 1 — Supporting Information Annex 1: Serum ferritin levels reference range according to age and sex. Modified from Soghier et al. [15]. [file MI-2026-8962790-s001.docx]

**Supplementary material**

| Age | Ferritin (ng/mL) | |
| --- | --- | --- |
| 4-<15 days | 99.6 – 717.0 | |
| 15 days-<6 months | 14.0 – 647.2 | |
| 6 months -<1 year | 8.4 – 181.9 | |
| 1 -<5 years | 5.3 – 99.9 | |
| 5 -<14 years | 13.7 – 78.8 | |
| 14- <16 years | Male: 12.7 – 82.8 | Female: 5.5 – 67.4 |
| 16 -<19 years | Male: 11.1 – 171.9 |  |

Annex 1. Serum ferritin levels reference range according to age and sex. Modified from Soghier LM, et al. Reference Range Values for Pediatric Care. Vol. 2nd edition. Itasca, IL: American Academy of Pediatrics; 2019.
